# A pilot proof-of-principle study to compare fresh and vitrified cycle preimplantation genetic screening by chromosome microarray and next generation sequencing

**DOI:** 10.1186/s13039-016-0238-8

**Published:** 2016-03-21

**Authors:** Gwo-Chin Ma, Hsin-Fu Chen, Yu-Shih Yang, Wen-Hsiang Lin, Feng-Po Tsai, Chi-Fang Lin, Chi Chiu, Ming Chen

**Affiliations:** Department of Genomic Medicine and Center for Medical Genetics, Changhua Christian Hospital, Changhua, Taiwan; Department of Genomic Science and Technology, Changhua Christian Hospital Healthcare System, Changhua, Taiwan; Institute of Biochemistry, Microbiology and Immunology, Chung-Shan Medical University, Taichung, Taiwan; Department of Medical Laboratory Science and Biotechnology, Central Taiwan University of Science and Technology, Taichung, Taiwan; Department of Obstetrics and Gynecology, College of Medicine and Hospital, National Taiwan University, Taipei, Taiwan; Graduate Institute of Medical Genomics and Proteomics, College of Medicine, National Taiwan University, Taipei, Taiwan; Poyuan Women Clinic, Changhua, Taiwan; Department of Medical Genetics, National Taiwan University Hospital, Taipei, Taiwan; Department of Obstetrics and Gynecology, Changhua Christian Hospital, Changhua, Taiwan; Department of Life Science, Tunghai University, Taichung, Taiwan

**Keywords:** Chromosomal microarray, Aneuploidy, Fresh embryo transfer, SET, PGS

## Abstract

**Background:**

Single embryo transfer (SET) has been utilized as a strategy to reduce the chance of multifetal gestations in in vitro fertilization (IVF) but lower pregnancy rate remains a concern. Recent studies showed that favorable outcome regarding SET can be achieved by selecting embryos with “more normal” genetic components. We explored the use of rapid array comparative genomic hybridization (aCGH) to select blastocysts for fresh SET and compared with the protocols adopting vitrified (ultrarapidly frozen) embryo transfer cycle. Validation of the rapid protocol of aCGH and comparison of the result with the regular protocol of aCGH and next generation sequencing (NGS) are also performed.

**Results:**

First-time IVF patients with normal karyotype (*n* = 21) were enrolled for elective fresh SET cycle (*n* = 8; designated as fresh SET group) or vitrified embryo transfer cycle (*n* = 13; designated as vitrified ET group) coupling with comprehensive chromosomal screening by a 9-h rapid aCGH from Day 5 trophectoderm (TE) biopsy. In fresh SET group, 86 blastocysts (10.8 blastocysts/patient) were biopsied and analyzed. Aneuploidy was detected in 53.5 % (46/86) of the biopsied blastocysts. All patients had a single embryo transferred on the following day. The clinical pregnancy rate was 87.5 % (7/8) and the ongoing pregnancy rate was 62.5 % (5/8). In vitrified ET group, 58 blastocysts (4.5 blastocysts/patient) were biopsied and 56 blastocysts were analyzed. Aneuploidy was detected in 39.3 % (22/56) of biopsies. The patients accepted for SET or double embryos transfer (DET) in non-stimulated cycles. The clinical pregnancy rate and the ongoing pregnancy rate was 76.9 % (10/13) and 53.8 % (7/13) respectively. Spontaneous abortions occurred in both of the two patient groups. In the series of fresh SET group, no twin pregnancy was noted and at least one healthy baby had been born at gestational age (GA) 37^+6^ weeks when submission. The results of PGS by rapid aCGH, regular aCGH and NGS were comparable in most occasions.

**Conclusion:**

This study evaluates the use of rapid aCGH to select blastocysts for fresh SET and demonstrates its feasibility in a real clinical IVF program. A successful livebirth is achieved and the favorable outcome is superior to the protocol adopting vitrified ET cycle in our own setting. Additional studies are needed to verify this pilot data and validate its application in large randomized trials.

## Background

Preimplantation genetic screening (PGS) has been considered a feasible strategy, by reducing the probability of transferring the aneuploid embryos during in vitro fertilization (IVF), in improving the implantation rate as well as the livebirth rate in the practice of artificial reproductive technology (ART) for decades [1]. However, a much wider acceptance by the academic community does not occur until recently, by some recent seminal studies which allowed us to have a more comprehensive understanding of the critical points in the whole IVF-PGS process [2–5]. The famous “Mastenbroek controversy” published in 2007 stating that for women with advanced maternal age, the livebirth rate of the PGS group was poorer than the non-PGS group did raise a huge concern for the validity of PGS and the traditional PGS by fluorescence in situ hybridization (FISH) was almost abandoned [6, 7]. However, more and more recent studies had made us known that the ironic outcome revealed by that randomized study may be due to the limitations of the genetic tool used for PGS itself (FISH), as well as the timing of the biopsy was set at the cleavage stage embryos (Day 3). The outcomes of PGS, especially the most important livebirth rate, are at least greatly affected by three critical parameters: the timing of biopsy, the genetic tools used for PGS, and to freeze or not to freeze the biopsied embryos [8–12]. After 2007, since the emergence of many new technologies available for genetic investigation, researchers are keen to verify and validate the new tools when being used in PGS [5, 13–15]. Nowadays it is a reality to use the latest technology such as array comparative genomic hybridization (aCGH) and next generation sequencing (NGS) platforms to screen the embryos because of the advancement of a critical technique called whole genome amplification (WGA). The aCGH and NGS had become accessible for PGS after WGA [15–19]. However, such amplification may also introduce errors and thereby other research groups had heartily advocated for WGA-free quantitative polymerase chain reaction (qPCR) based rapid diagnosis [8, 11, 20]. Excellent results were achieved by even using single embryo transfer (SET) in fresh cycle by the group led by Richard Scott Jr and Nathan Treff based in US [11], and even with a limited set of chromosomes being tested by our group [21].

It is now understood that the blastomere biopsy at Day 3 cleavage-stage embryos may impair the implantation potential while the trophectoderm (TE) biopsy at Day 5/6 blastocysts may not [3]. However, due to the sophistication of the genetic tools used to screen the developing embryos, it is not always feasible to have the report turnout time to be less than one day, since all embryos must not survive in vitro after 7 days post-fertilization unless being frozen. Therefore in before for all amplification-based technologies, including aCGH and NGS, it is not feasible to do fresh embryo transfer if the timing of the biopsy was set at Day 5/6. Earlier studies did report some cycles based upon PGS by aCGH on Day 3 embryos [10, 22–24], but very few at Day 5/6 because the protocol of aCGH usually needs at least 2–3 days, in which the time for analysis was not counted in [25]. To freeze the biopsied embryos sent for PGS, and to transfer the thawed euploid embryos in non-stimulated cycles, becomes the standard practice for most IVF-PGS centers. Recently, some researchers showed that the combination of PGS and embryo vitrification (ultrarapid freeze) can produce better clinical outcomes compared to non-PGS ET [26] while we considered fresh ET remains a preferred choice in routine IVF if a rapid PGS is feasible. Quite recently the newest protocol of rapid aCGH analyzes suited for PGS was available, and therefore, we conducted this pilot study to evaluate its feasibility in a real clinical setting: TE biopsy on Day 5, 9-h aCGH analysis, followed by fresh SET on Day6. Additionally, we compared the results with the protocols adopting vitrified ET cycle.

## Results

### Rapid PGS aCGH

**Fig. 1:**
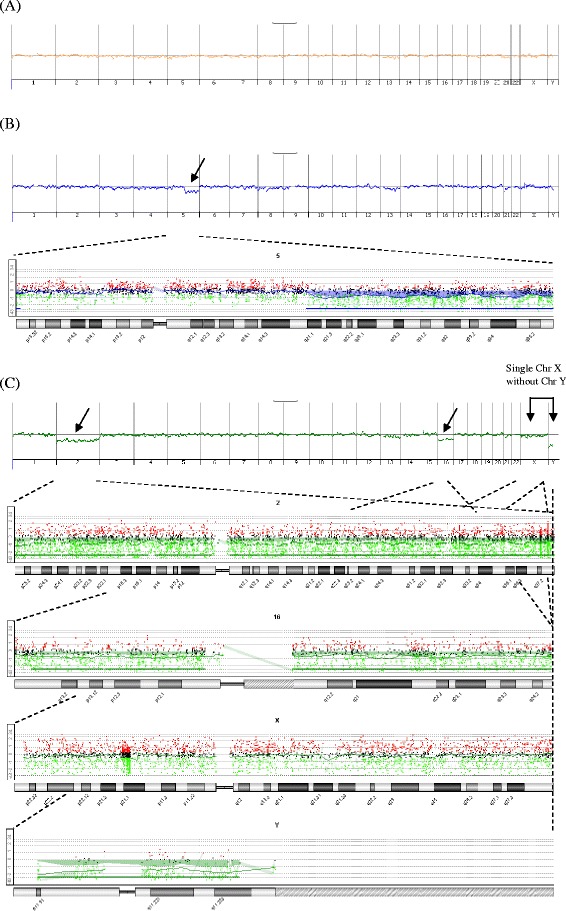
Representative rapid aCGH results of blastocysts obtained by trophectoderm biopsy at post-fertilization Day5 showed **a** euploid chromosomes [arr(1–22) × 2,(XY) × 1], **b** a segmental aneuploidy [arr 5q15q35.3(94,800,050-180,684,501) × 1] and **c** a complex chromosomal abnormality [arr(1,3-15,17-22) × 2,(2) × 1,(16) × 1,(X) × 1]. Arrow indicated the aneuploidy chromosome or chromosomal fragment. The reference DNA used for aCGH was whole genome amplification product of a normal male embryo DNA

**Fig. 2:**
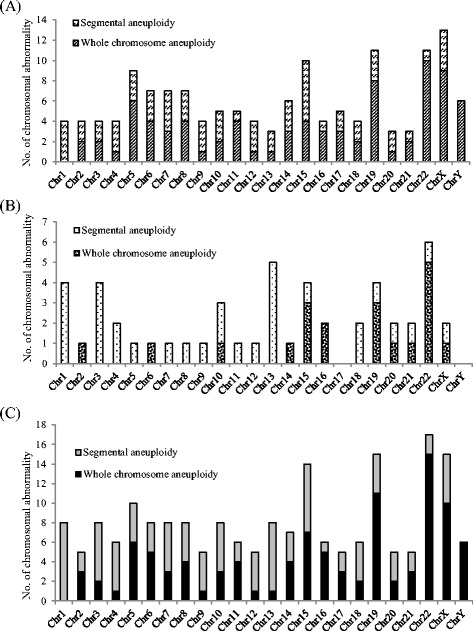
Chromosomal abnormalities, at the single-chromosome level, detected by rapid aCGH in **a** 86 embryos from 8 patients accepted for fresh SET, **b** 58 embryos from 13 patients choosing vitrified ET, and **c** all 144 embryos from the 21 patients. A total of 194 chromosome abnormalities (143 in in fresh SET group and 51 in vitrified ET group), including 102 whole chromosome gain/loss and 92 segmental aneuploidies, were found in 68 aneuploid embryos

**Fig. 3:**
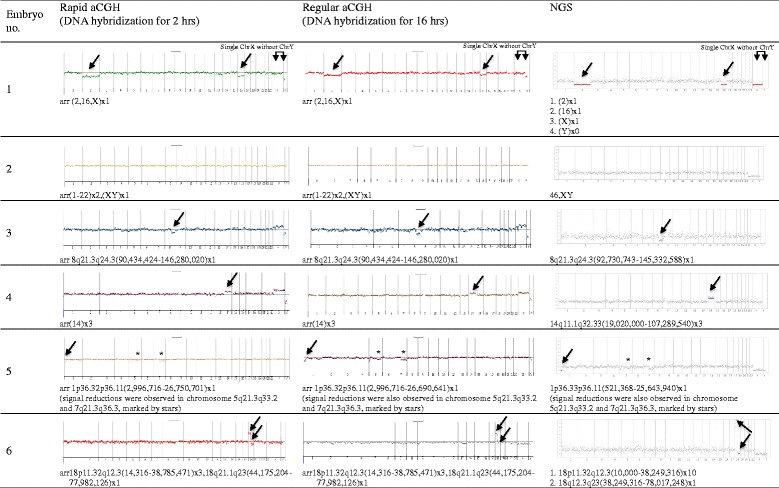
Exemplified PGS results by use of rapid aCGH (DNA hybridization for 2 h), regular aCGH (DNA hybridization for 16 h) and next generation sequencing (NGS) for the same WGA products. Rapid and regular aCGH were performed with CytoScan 60 K microarray chip (Agilent customer array, Changhua Christian Hospital, Taiwan) on a G4900DA SureScan microarray scanner (Agilent Technologies, CA, USA). NGS was performed using Ion PGM Hi-Q Sequencing Kit with Ion 316 chip (Life technologies, California, USA) on the Ion Torrent PGM Instrument (Life technologies) platform. Aneuploidy chromosomes or chromosomal fragments are indicated by arrows. Some atypical segmental gains and/or losses with copy number change < 1 but > 0.5 (a likely result of embryo mosaicism) were also classified as segmental aneuploidies and marked by stars. The results of rapid aCGH are comparable with that of regular aCGH and NGS

A total of 21 patients were subjected to in IVF-rapid PGS aCGH treatment, including 8 patients with fresh SET (fresh SET group) and 13 patients with vitrified SET/DET (vitrified ET group) (Table 1). The average age of the patients was 36.0 years (range of 29–42 years), with a mean age of 33.6 years (range of 29–37 years) in fresh SET group and 37.5 years (range of 34–42 years) in vitrified ET group. In fresh SET group, 86 blastocysts (10.8 blastocysts/patient) were biopsied and analyzed. Euploidy was found in 46.5 % (40/86) of the embryos, whereas chromosome abnormalities were found in the remaining 53.5 % (46/86) of embryos (Table 1). Among the aneuploid blastocysts, 43.5 % (20/46) had single chromosomal abnormality [of which. 35.0 % (7/20) displayed single chromosome loss, 15.0 % (3/20) displayed single chromosome gain and 50.0 % (10/20) displayed single segmental aneuploidy with an aberration between 6.4 Mb and 85.9 Mb], 21.7 % (10/46) had dual chromosomal abnormalities and 34.8 % (16/46) had severe, compound genetic defects involving in three or more chromosomes (Table 2 and Fig. 1). Look at the single-chromosome level, 143 chromosome abnormalities, including 82 whole chromosome gain/loss and 61 segmental aneuploidies, were found in the 46 aneuploid embryos (Fig. 2a). In vitrified ET group, a total of 58 blastocysts (4.5 blastocysts/patient) were biopsied. Of which, two blastocysts failed with WGA and thus finally 56 blastocysts were analyzed. Euploidy and aneuploidy were detected in 60.7 % (34/56) and 39.3 % (22/56) of biopsies respectively (Table 1). Among the aneuploid blastocysts, 59.1 % (13/22) had single chromosomal abnormality [of which. 30.8 % (4/13) displayed single chromosome loss, 15.4 % (2/13) displayed single chromosome gain and 53.8 % (7/13) displayed single segmental aneuploidy with an aberration between 12.3 Mb and 75.4 Mb], 13.6 % (3/22) had dual chromosomal abnormalities and 27.3 % (6/22) had severe, compound genetic defects involving in three or more chromosomes (Table 2). In the single-chromosome level, 51 chromosome abnormalities, including 20 whole chromosome gain/loss and 31 segmental aneuploidies, were found in the 22 aneuploid embryos (Fig. 2b). Overall, the aneuploidy rate of the 142 embryos examined was 47.9 % (68/142). Chromosome abnormalities were detected in all chromosomes where genetic defects involving chromosome 5, 15, 19, 22 and X were most frequently observed while errors in chromosome 2, 9, 12, 17, 20 and 21 seen relatively uncommon (Fig. 2c). The diagnostic results were validated by regular aCGH and NGS (see the *Methods* section and Fig. 3). The rate of correct diagnosis was 100 % in all embryos. The positive and negative predictive rates of rapid aCGH in our series were thus 100 %.

**Table 1:** Summary of rapid aCGH results for 86 embryos from 8 patients accepted for fresh single embryo transfer (SET) and 58 embryos from 13 patients choosing vitrified SET or double embryo transfer (DET)

Case no.	Age of patient	No. of embryos for aCGH				SET or DET	Chemical pregnancy (+hCG)	Outcome
		Total	WGA failure	Euploid	Aneuploid			
I. Fresh SET								
1	29	10	0	1	9	SET	Yes	Live birth^a^ (at GA = 37^+6^ weeks)
2	33	9	0	4	5	SET	Yes	Abortion (at GA = 5 weeks)
3	34	14	0	3	11	SET	No	—
4	34	14	0	6	8	SET	Yes	Ongoing pregnancy (GA = 37 weeks)
5	37	13	0	7	6	SET	Yes	Ongoing pregnancy (GA = 13 weeks)
6	34	9	0	5	4	SET	Yes	Abortion (at GA = 4 weeks)
7	37	9	0	8	1	SET	Yes	Ongoing pregnancy (GA = 16 weeks)
8	31	8	0	6	2	SET	Yes	Ongoing pregnancy (GA = 7 weeks)
Overall		86	0	40	46			
II. Vitrified ET								
1	41	3	0	1	2	SET	Yes	Live birth (at GA = 39 weeks)
2	40	4	0	1	3	SET	Yes	Termination (acardiac monster) (at GA = 14^+4^ week)
3	34	4	0	3	1	SET	Yes	Ongoing pregnancy (GA = 19^+4^ week)
4	39	5	0	1	4	SET	Yes	Ongoing pregnancy (GA = 22 week)
5	42	3	0	2	1	DET	No	—
6	37	6	0	5	1	DET	Yes	Live birth (at GA = 38 weeks)
7	36	5	0	4	1	DET	Yes	Live birth (at GA = 37 weeks)
8	37	5	0	2	3	DET	Yes	Abortion (at GA = 8^+3^ weeks)
9	42	5	0	2	3	DET	No	—
10	37	3	0	3	0	DET	Yes	Ongoing pregnancy (GA = 16 week)
11	34	5	0	5	0	DET	No	—
12	34	5	0	3	2	DET	Yes	Abortion (at GA = 10^+6^ weeks)
13	35	5	2	2	1	DET	Yes	Ongoing pregnancy (GA = 12^+3^ weeks)
Overall		58	2	34	22			

^a^Single birth (3040 g; AS 8 > 9) by C-section due to breech presentation

*WGA* whole genome amplification, *GA* gestational age

**Table 2:** Details of rapid aCGH results of aneuploid blastocysts derived from 8 patients for fresh SET and 13 patients for vitrified ET

Case no.	Total no. of aneuploid blastocysts	Single chromosomal abnormality [Single chromosome loss/Single chromosome gain/Single segmental aneuploidy]	Dual chromosomal abnormality	Complex chromosomal abnormality
I. Fresh SET				
1	9	4 (44.4 %) [1/2/1]	2 (22.2 %)	3 (33.3 %)
2	5	3 (60.0 %) [1/1/1]	0 (0 %)	2 (40.0 %)
3	11	2 (18.2 %) [1/0/1]	5 (45.5 %)	4 (36.4 %)
4	8	3 (37.5 %) [0/0/3]	1 (12.5 %)	4 (50.0 %)
5	6	4 (66.7 %) [3/0/1]	0 (0 %)	2 (33.3 %)
6	4	2 (50.0 %) [0/0/2]	2 (50.0 %)	0 (0 %)
7	1	1 (100.0 %) [0/0/1]	0 (0 %)	0 (0 %)
8	2	1 (50.0 %) [1/0/0]	0 (0 %)	1 (50.0 %)
Overall	46	20 (43.5 %) [7/3/10]	10 (21.7 %)	16 (34.8 %)
II. Vitrified ET				
1	2	1 (50.0 %) [1/0/0]	0 (0 %)	1 (50.0 %)
2	3	2 (66.7 %) [0/0/2]	1 (33.3 %)	0 (0 %)
3	1	1 (100.0 %) [0/0/1]	0 (0 %)	0 (0 %)
4	4	2 (50.0 %) [1/1/0]	1 (25.0 %)	1 (25.0 %)
5	1	1 (100.0 %) [0/1/0]	0 (0 %)	0 (0 %)
6	1	1 (100.0 %) [0/0/1]	0 (0 %)	0 (0 %)
7	1	1 (100.0 %) [0/0/1]	0 (0 %)	0 (0 %)
8	3	2 (66.7 %) [0/0/2]	0 (0 %)	1 (33.3 %)
9	3	1 (33.3 %) [1/0/0]	1 (33.3 %)	1 (33.3 %)
10	0	0 (0 %) [0/0/0]	0 (0 %)	0 (0 %)
11	0	0 (0 %) [0/0/0]	0 (0 %)	0 (0 %)
12	2	1 (50.0 %) [1/0/0]	0 (0 %)	1 (50.0 %)
13	1	0 (0 %) [0/0/0]	0 (0 %)	1 (100.0 %)
Overall	22	13 (59.1 %) [4/2/7]	3 (13.6 %)	6 (27.3 %)

### Clinical outcome

In both of fresh SET and vitrified ET group, all patients had at least one euploid embryo available for fresh transfer on Day 6 (fresh SET group) or in non-stimulated cycles (vitrified ET group). Combining the data of rapid aCGH and blastocyst grading, the best-graded euploid embryo(s) were chosen for ET for each of the patients. In fresh SET group, the clinical pregnancy rate was 87.5 % (7/8) and the ongoing pregnancy rate was 62.5 % (5/8). Spontaneous abortions occurred in two patients at GA 5 and GA 4 weeks (the genetic analysis cannot be performed due to the spontaneous expulsion of the gestational tissue). No twin pregnancy was noted in this series and at least one healthy baby had been born at GA 37^+6^ weeks (Table 1). In vitrified ET group, the clinical pregnancy rate and the ongoing pregnancy rate was 76.9 % (10/13) and 53.8 % (7/13) respectively. Spontaneous abortions occurred in two patients at GA 8^+3^ and GA 10^+6^ weeks. One fetus was terminated at GA 14^+4^ weeks due to acardiac monster. Genetic analysis by aCGH for tissues from the aborted and terminated fetuses revealed no specific finding; all cases had a normal karyotype.

## Discussion

PGS has become a well-established tool in the practice of IVF nowadays with the development of many sophisticated tools used for investigating the genetic complements of the embryos before being transferred [14, 27]. However, the major difficulty in these genetic tools, such as the latest NGS, is the complexity of analytical pipelines [28]. Therefore to freeze the embryos to earn more time for the reliable analysis of the PGS results remains the mainstream in most PGS centers, especially those involving aCGH and NGS [19]. It is now known that the biopsy timing will be better at Day 5/6 blastocyst stage instead of Day 3 cleavage-stage embryos in regard to the implantation potential [3], and this new finding creates an even more difficulty: the time span of analysis if we want to do it in fresh transfer will be shortened from 3 days to 1 day, which rendered the analysis of aCGH and NGS almost impossible. Up to this submission, there are very few reports achieving this goal when using high-complexity-tool such as aCGH or NGS and also successfully conducted the fresh SET [25, 29].

In order to facilitate the analytical bioinformatics pipelines used in aCGH and NGS, some resolution was sacrificed for efficiency and also because of the process of WGA may introduce errors, and some leading groups admitted it is now available for PGS to use these fancy technologies in a “low-pass” strategy [18].

It is now well known that mosaicism is a common phenomenon in the development of early human embryos [30], and therefore whether the “best” embryo can really be selected with the most sophisticated genetic tools remains in doubt. Another group thus proposed for amplification-free quantitative polymerase chain reaction (qPCR) based PGS strategy, which only detects the copy number changes involving the whole chromosomal arm level, and surprisingly the result was excellent. They published a series of excellent data to demonstrate that by using qPCR, the accuracy was comparable to aCGH [8, 15, 20], the implantation rate and delivery rate were also excellent [2, 4], and such impression even was validated in a randomized trial concerning delivery rate and neonatal/obstetrical outcome (the BEST trial) [11].

Regarding the aneuploidies detected by rapid aCGH in this pilot study, the overall aneuploidy rate was 47.9 % (53.5 % in fresh SET group and 39.3 % in vitrified ET group), which is similar to a recent series comparing aCGH and NGS (which is around 60 %, see Yang et al., 2015 [19]), and was considered to be a result that both of the analyses included segmental aneuploidies greater than 5 Mb (in Yang’s series, they reported a 42 Mb gain on 16q and a 16 Mb loss on 18q). The cytogenetic abnormalities distributed in all 24 chromosomes. However, in a recent validation study to compare NGS and aCGH to detect the cytogenetic abnormalities in the TE cells of human embryos, the resolution has been greatly enhanced to 1.19-3.89 Mb [31]. In theory the resolution of aCGH and NGS must be much higher than qPCR (which only detects aneuploidy in a chromosomal arm level and the aneuploidy rate was around 30 %, see Forman et al., 2014 [11]), however, it has been noted that aCGH may have a higher false positive rate than qPCR [20], in which some euploid embryos may be sacrificed and not transferred. Our report is one of the very few studies up to 2016 (and the first report from Taiwan) adopting Day 5 blastocysts biopsy, 9-h aCGH and fresh SET to achieve successful singleton livebirth. Furthermore, the favorable outcome in terms of the clinical pregnancy rate and the ongoing pregnancy rate is superior to the protocol adopting vitrified ET cycle (87.5 and 62.5 % in fresh SET group, and 76.9 % and 53.8 % in vitrified ET group). We admit the case number is very small (*n* = 8 in fresh SET group and *n* = 13 in vitrified ET group) and it is only a pilot study which needs further validation in a prospective, large-scale and better be randomized study to explore its real efficacy.

However, despite in theory the resolution of NGS should be much better than aCGH in picking up aberrations in genetic complements. The current “low-pass strategy” adopted by NGS made it only comparable to the aCGH platform when using in PGS [19, 31, 32]. With the advance of bioinformatics pipelines it can be expected that NGS can pick up more aneuploidies than aCGH, as well as the turn-around time for analysis should be greatly shortened, to make fresh embryo transfer feasible in the near future. When three competing PGS tools (qPCR, aCGH and NGS) can all produce results in one single day, then it is possible to conduct a real randomized trial to compare the efficacy and may further enhance our understanding of the early development of human embryos, and to improve the whole IVF-PGS process.

It is noteworthy that despite it was thought that freeze-thaw process might damage the embryos whereas a growing number of epidemiological studies had suggested otherwise: that an increased rate of adverse perinatal outcomes such as low birth weight was noted in fresh IVF cycles compared with frozen embryo transfer cycles. It is considered superovulation has a significant impact upon endometrial receptivity and therefore frozen embryo transfer cycles should be considered in those high-responders [33]. However, we considered that if in the future time constraint is no longer considered a problem when the advantage and safety of frozen ET cycles are well established [34], the effort should be placed upon improving the bioinformatics pipelines of NGS from “low-pass-strategy” into an algorithm with a better resolution that can better utilize the advantage of NGS technology itself.

## Methods

### Patient recruitment

During January of 2014 to December of 2015, 21 infertile couples visited the clinic center for their first-time IVF and elected either fresh SET (*n* = 8; designated as fresh SET group) or vitrified SET/DET (*n* = 13; designated as vitrified ET group) by conjugation with comprehensive chromosomal screening via a 9-h rapid PGS aCGH protocol for trophectoderm (TE) biopsy on Day 5. All the couples have a normal karyotype and the patients are expected to have a good prognosis (female age ≤ 42 years, no prior miscarriage and no remarkable personal and family history). After pre-treatment counseling was provided, all the 21 couples agreed to participate in this study and signed an informed consent prior to the sample collection and evaluation. The study was approved by the Ethics Committees of Changhua Christian Hospital, Changhua, Taiwan and adhered to the guidelines approved by the Institutional Review Board.

### Fertilization, embryo culture and TE biopsy

Intracytoplasmic sperm injection (ICSI) was performed following the removal of cumulus cells and the presence of two pronuclei and two polar bodies 16–18 h after injection was ascertained normal fertilization. Fertilized embryos were cultured in sequential media (Sage, CA, USA) to the blastocyst stage. On Day 3, a laser-created narrow channel was made in the zona pellucida of all embryos. On Day 5, fully differentiated embryos were biopsied using suction to gently extrude and pull 3–8 TE cells from blastocysts. The biopsied cells were washed in 1X PBS (catalog no. 70013–032) (Gibco by life technology, CA, USA) and collected into a microcentrifuge tube with 2.5 μl 1X PBS (Gibco by life technology). The cells were used directly for whole genome amplification (WGA).

### Rapid aCGH

The WGA was performed using REPLI-g Single Cell Kit (Qiagen, Hilden, Germany) and following the manufacturer’s instructions. Amplified DNA was purified using the QIAamp DNA Blood Mini Kit (Qiagen). The DNA purities and concentrations were examined by Nanodrop 2000 spectrophotometer (Thermo Fisher Scientific, Delaware, USA). Approximately 1 μg of purified DNA was fluorescently labeled with Cy3 d-CTP or Cy5-dCTP using SureTag DNA Labeling Kit (Agilent Technologies, CA, USA) and then cleaned up by Microcon YM-30 centrifugal filter unit (Millipore, MA, USA). The yield DNA was hybridized with CytoScan 60 K microarray chip (Agilent customer array, Changhua Christian Hospital, Taiwan) at 65 °C for 2 h. The image on a chip was acquired with a G4900DA SureScan microarray scanner (Agilent Technologies, CA, USA) and analyzed with Agilent Genomic Workbench software (Agilent Technologies) for DNA gain or loss across all 24 chromosomes. Aberrations were detected by using default setting with an algorithm of z-score conjugated with a filter of a minimum of 5 Mb aberrations. All aCGH procedures were completed within 9 h, allowing rapid evaluation of the TE biopsies and providing a feasible opportunity for fresh embryo transfer on the following day, Day 6.

### Regular aCGH

To evaluate the reliability of rapid aCGH, WGA products of all the biopsied blastocysts were processed for the regular aCGH analysis. All procedures of regular aCGH analysis are the same as that described in rapid aCGH, except for the DNA-chip probes hybridization duration which was extended (from 2 h) to 16 h.

### NGS

WGA products of all the biopsied blastocysts were also processed for the NGS analysis in order to evaluate the reliability of rapid aCGH and compare the performances of difference platforms (i.e. rapid aCGH, regular aCGH and NGS). Approximately 1 μg of WGA DNA was used for library construction using Ion Xpress Plus gDNA Fragment Library Preparation Kit Set (Life technologies, California, USA) and following the manufacturer’s instructions. The quantity of library was determined using Qubit dsDNA HS assay kits (Life technologies) with Qubit fluorometer (Life technologies). The template-positive Ion Sphere Particles (ISPs) were generated using Ion PGM Hi-Q Template Kits (Life technologies) with the Ion OneTouch 2 Instrument (Life technologies) and then enriched with the Ion OneTouch ES Instrument (Life technologies). Sequencing was performed on the Ion Torrent PGM Instrument (Life technologies) platform using the Ion PGM Hi-Q Sequencing Kit and Ion 316 chip (Life technologies). PGS analysis for aneuploidy detection was performed by the Ion Reporter Server System (https://ionreporter.thermofisher.com/ir/).

### Embryo selection for transfer

Only euploid blastocyst ascertained by rapid aCGH analysis was selected for transfer. When multiple euploid blastocysts are available, the best grade one was chosen for transfer. Embryo grading is based on the principle proposed in Sakkas and Gardner (2005) [35]. In brief, the blastocyst is given a grade based on three main components of the embryo: (1) expansion degree and hatching status, (2) the inner cell mass (ICM) development, and (3) the TE cell quality. The graded embryo is given a number grade (1–6), followed by a letter grade for the ICM and then the TE (A, B or C). All embryos, which were classified as aneuploid, were confirmed again by aCGH before discarded.

## Conclusions

In this study, we demonstrate the results of aCGH (including rapid and regular protocol) and NGS (regular protocol) being used for PGS are comparable to each other with high consistency. Furthermore, we also demonstrate the feasibility of using rapid protocol aCGH to select blastocysts for fresh SET in a real clinical IVF setting, in which at least one successful live-birth has already been achieved. Meanwhile, in terms of the clinical pregnancy rate as well as the ongoing pregnancy rate, PGS by aCGH followed by fresh cycle ET seems superior (or at least comparable) to PGS by aCGH coupled with vitrified cycle ET. Additional studies are needed to verify this pilot data and validate its clinical utility in large prospective randomized trials.

## Abbreviations

aCGH: array comparative genomic hybridization
ART: artificial reproductive technology
DET: double embryo transfer
ET: embryo transfer
FISH: fluorescence in situ hybridization
GA: gestation age
ICM: inner cell mass
ICSI: intracytoplasmic sperm injection
ISPs: ion Sphere Particles
IVF: in vitro fertilization
NGS: next generation sequencing
PGS: preimplantation genetic screening
qPCR: quantitative polymerase chain reaction
SET: single embryo transfer
TE: trophectoderm
WGA: whole genome amplification
